# *Ceratocorys mariaovidiorum* sp. nov. (Gonyaulacales), a new dinoflagellate species previously reported as *Protoceratium reticulatum*

**DOI:** 10.1111/jpy.12609

**Published:** 2017-12-29

**Authors:** Pablo Salgado, Santiago Fraga, Francisco Rodríguez, Pilar Riobó, Isabel Bravo

**Affiliations:** ^1^ División de Investigación en Acuicultura Departamento de Medio Ambiente Instituto de Fomento Pesquero (IFOP) Enrique Abello 0552, Casilla 101 Punta Arenas Chile; ^2^ Centro Oceanográfico de Vigo Instituto Español de Oceanografía (IEO) Subida a Radio Faro 50 36390 Vigo Spain; ^3^ Instituto de Investigaciones Marinas (IIM‐CSIC) Eduardo Cabello 6 36208 Vigo Spain

**Keywords:** *Ceratocorys mariaovidiorum*, dinoflagellate, Gonyaulacales, ITS rDNA, *Protoceratium reticulatum*, reticulated plates

## Abstract

The family Ceratocoryaceae includes the genera *Ceratocorys*,* Protoceratium,* and *Schuettiella*, whose phylogenetic relationships are poorly known. Here, the new non‐yessotoxin‐producing species of the genus *Ceratocorys*,* Ceratocorys mariaovidiorum* sp. nov., previously reported as the toxic *Protoceratium reticulatum*, is described from examinations by light and scanning electron microscopy, molecular phylogeny, and toxin analyses. The species description is made from culture samples of strains CCMP1740 and CCMP404 from USA waters. *Ceratocorys mariaovidiorum* is globular and has thick and strongly reticulated plates with one pore within each reticule, just like *P. reticulatum*, but the key difference between the two species is the presence of five precingular plates in *C. mariaovidiorum* instead of six as in *P. reticulatum*. The thecal plate formula is Po, 4′, 0a, 5″, 6c, ~7s, 5‴, 0p, 2′′′′. The apical pore plate is oval with a λ‐shaped pore. The first apical plate is narrow with a ventral pore on the right anterior side; it contacts the apical pore plate and its contact with the anterior sulcal plate is slight or absent. The fourth precingular plate of other Gonyaulacales is absent. *Ceratocorys mariaovidiorum* may have small spines on the second antapical plate. A phylogenetic study based on internal transcribed spacer/5.8SrDNA supports the morphological classification of *C. mariaovidiorum* as a new species of *Ceratocorys* and in a different clade from *P. reticulatum*.

**Abbreviations**
AIF: all ion fragmentation
ITS rDNA: internal transcribed spacer rDNA
L:D: light:dark
LC–HRMS: liquid chromatography coupled to high‐resolution mass spectrometry
NCMA: National Center for Marine Algae and Microbiota
YTX: yessotoxin

*Ceratocorys* is a genus of marine dinoflagellates belonging to the order Gonyaulacales and to the family Ceratocoryaceae (Lindemann 1928), which also includes the genera *Protoceratium* and *Schuettiella* (Gómez 2012). The genus *Ceratocorys* currently comprises 12 taxonomically accepted species (*C. anacantha*,* C. armata*,* C. aultii*,* C. bipes*,* C. gourretii*,* C. grahamii*,* C. horrida*,* C. indica*,* C. kofoidii*,* C. magna*,* C. reticulata*, and *C. skogsbergii*), among which the most representative is *C. horrida*, its type species (Guiry 2017). All its members share characteristics such as being oceanic, inhabiting warm waters in tropical and subtropical regions worldwide, and building a cell wall of cellulosic plates (Graham 1942, Balech 1988, Fensome et al. 1993, Carbonell‐Moore 1996, Steidinger and Tangen 1997). Most species are phototrophic. Hallegraeff and Jeffrey (1984) mentioned that *C. horrida* showed intensely pigmented red fluorescent cells in shelf waters and non‐pigmented green fluorescent cells at offshore stations, while *C. armata* only displayed green fluorescence typical of non‐photosynthetic dinoflagellates. To our knowledge, no toxin studies of this genus have been carried out, so nothing is known about their toxicity. They all have a striking ornamentation and a distinctive general morphology. In general, they have a well‐sculpted theca, a flat epitheca commonly much shorter than the hypotheca, broad cingular lists or crests, and short or large hypothecal spines (four projecting from the edges of the antapical plate and two single dorsal and ventral spines; Fensome et al. 1993, Zirbel et al. 2000). Until now the only two described species that do not have spines are *C. anacantha* and *C. grahamii* (Carbonell‐Moore 1996). Conversely, the main differences reported among these species are in their cell size, ornamentation (which can be reticulated or smooth, with high or low ridges), pore size, hypothecal spine length, and body shape.

The tabulation of thecal plates of *Ceratocorys* does not completely coincide with that of other gonyaulacoids. In the epitheca, it was described as having three apical plates, an intercalary plate (1a), and five precingular plates. In the hypotheca, it has five postcingular plates and two antapical plates (Graham 1942, Balech 1988, Fensome et al. 1993). The main feature differentiating it from other gonyaulacoid genera, such as *Alexandrium*,* Protoceratium*, and *Gonyaulax*, is the presence of five precingular plates instead of the six plates that the others have. The fourth precingular plate in *Ceratocorys* represents the fourth and fifth precingular plates of other Gonyaulacales, which means that they have fused or that one is missing (Fensome et al. 1993).

Here, we describe a new species of *Ceratocorys*,* Ceratocorys mariaovidiorum*, based on morphological, molecular phylogenetic, and toxin studies of the strains CCMP404 and CCMP1720, which had been previously reported as *Protoceratium reticulatum* (NCMA 2017). The considerable morphological similarity between the two species had led the distinctive characteristics of those strains to be overlooked. However, previous molecular analyses (Howard et al. 2009, Scorzetti et al. 2009, Akselman et al. 2015) using strains CCMP404, CCMP1720, CCMP1721, and other *P. reticulatum* strains had already noted a phylogenetic divergence between them. Moreover, the phylogenetic relationship had been mentioned by Gómez et al. (2011), who showed in their phylogenetic tree of maximum likelihood of SSU rDNA sequences that *Protoceratium reticulatum* and *Ceratocorys horrida* have evolved from a common ancestor.

## Materials and Methods

### Source of specimens and culture conditions

Strains CCMP404 and CCMP1720, listed as *Protoceratium reticulatum*, were obtained from the National Center for Marine Algae and Microbiota (NCMA, ME, USA) and kept in culture at the Culture Collection of Harmful Microalgae of the Instituto Español de Oceanografía in Vigo (CCVIEO; http://www.vgohab.es/). Strain CCMP404 was isolated from a hypersaline lake (Salton Sea) in California USA (33°22′ N, 116°0′ W) and strain CCMP1720 from Biscayne Bay, Miami, FL, USA (25°48′ N, 80°19′ W). The cultures were maintained in Erlenmeyer flasks filled with 50 mL of L1 medium (Guillard and Hargraves 1993) without silicates, prepared with Atlantic seawater from off the Ría de Vigo (Spain), adjusted to a salinity of 32 by the addition of sterile bi‐distilled water and maintained at 19°C ± 1°C and a photoperiod cycle of 12:12 h light:dark (L:D) with a photon irradiance of about 100 μmol photons · m^−2^ · s^−1^ of PAR measured with a QSL‐100 irradiance meter (Biospherical Instruments Inc., San Diego, CA, USA).

### Light microscopy

The morphological study of the strains by light microscopy was carried out in wells of culture plates (35 mm diameter; Thermo Fisher Scientific, San Jose, CA, USA) inoculated from the cultures maintained in Erlenmeyer flasks. For a detailed study of the thecal plates, vegetative cells were stained with Calcofluor white (Fluorescent Brightener 28; Sigma–Aldrich, St. Louis, MO, USA; Fritz and Triemer 1985) and observed under a Leica DMLA microscope (Leica Microsystems GmbH, Wetzlar, Germany) equipped with differential interference contrast, UV epifluorescence with UV and blue excitation filters, and an AxioCam HRc camera (Zeiss, Göttingen, Germany). When required, cells were dissected by squashing them using gentle pressure on the coverslip, occasionally with the aid of sodium hypochlorite. The cultured cells were observed alive or fixed with formaldehyde at a final concentration of 1%. Cell nuclei were stained with 1:100 SYBR Green in PBS 0.01 M pH 7 for 20 min and observed using the same microscope at 365 nm. Photographs were taken with an AxioCam HRc camera (Zeiss, Göttingen). When the depth of field was not sufficient for the whole object, several pictures were taken at a series of different foci and automatically merged using Adobe Photoshop (Adobe Systems Incorporated, San Jose, CA, USA).

Sexual stages such as gamete pairs (cells smaller and lighter in color than vegetative cells which are joined by epitheca) and planozygotes (longitudinally biflagellated cells; Salgado et al. 2017a) from strains cultured in L1 medium were observed under an inverted microscope (Axiovert Zeiss 135, Jena, Germany), video‐recorded, and photographed using a microscope camera (Canon EOS 5D Mark II, Tokyo, Japan). Cyst‐like cells were individually isolated (*n* = 5) and transferred into the wells of culture plates (8 mm diameter; Thermo Fisher Scientific) containing 250 μL of fresh L1 medium. The cyst‐like cells were incubated at 19°C ± 1°C with a photoperiod cycle of 12:12 h L:D (photon irradiance of about 100 μmol photons · m^−2^ · s^−1^) and examined daily for 15 d under an inverted microscope (Axiovert Zeiss 135, Jena). Photographs were obtained using a microscope camera (Canon EOS 5D Mark II, Tokyo, Japan).

### Scanning electron microscopy

For scanning electron microscopy (SEM), 3 mL samples of cultures with exponentially growing vegetative cells were fixed with glutaraldehyde at a final concentration of 4%. After 24 h at room temperature, the fixed cells were filtered through 5 μm pore size Isopore RTTP polycarbonate filters (Merck Millipore, Billerica, MA, USA), stained with 2% osmium tetroxide for 30 min, rinsed three times with distilled water, and dehydrated in a series of 30%, 50%, 70%, 90%, 95%, and 100% EtOH. They were then air‐dried overnight, coated with gold using a K550 X sputter coater (Emitech Ltd., Ashford, Kent, UK), and observed with an FEI Quanta 200 scanning electron microscope (FEI Company, Hillsboro, OR, USA).

### Plate nomenclature

**Figure 1:**
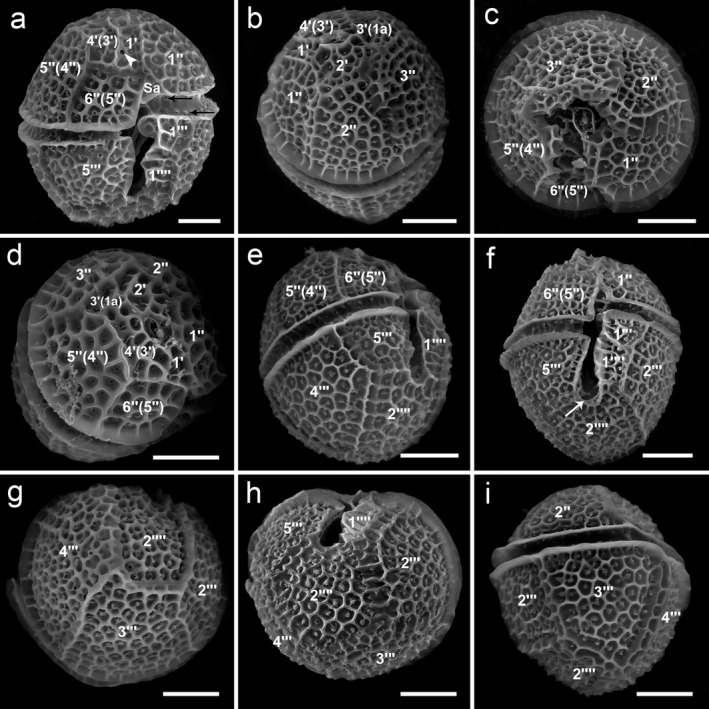
Scanning electron microscopy images of *Ceratocorys mariaovidiorum* sp. nov. cells. (a) Ventral view (arrowhead, ventral pore; arrows, pores in cingulum plates). (b) Dorsal‐apical view. (c) Apical view. (d) Lateral‐apical view. (e) Lateral‐antapical view. (f) Ventral view (arrow: notch). (g) Dorsal‐antapical view. (h) Antapical view. (i) Lateral view. Scale bar: 10 μm.

**Figure 2:**
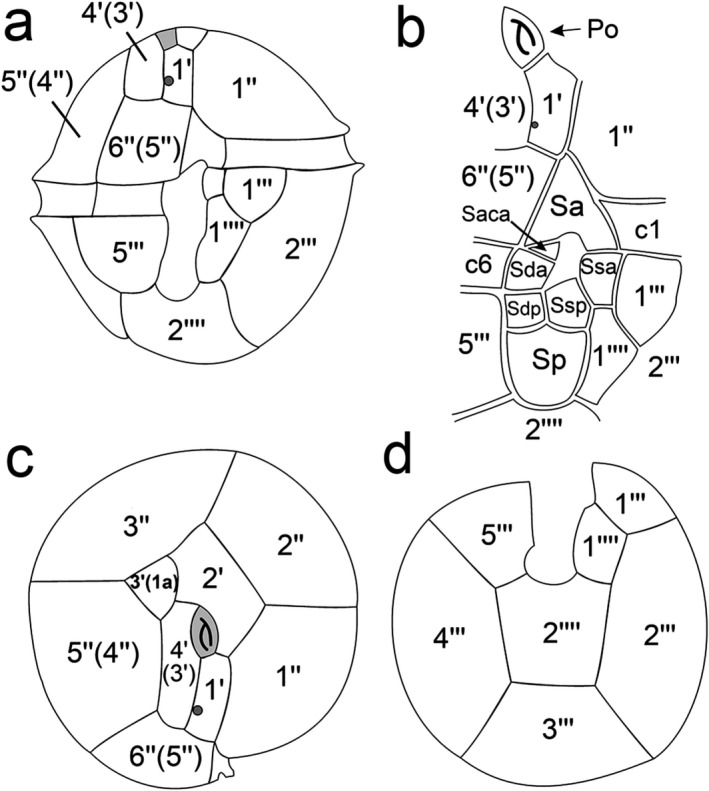
Schematic drawings of thecal plate patterns of *Ceratocorys mariaovidiorum* sp. nov. (a) Ventral view. (b) Sulcal area. (c) Apical view. (d) Antapical view. For the abbreviations see Table 1.

**Figure 3:**
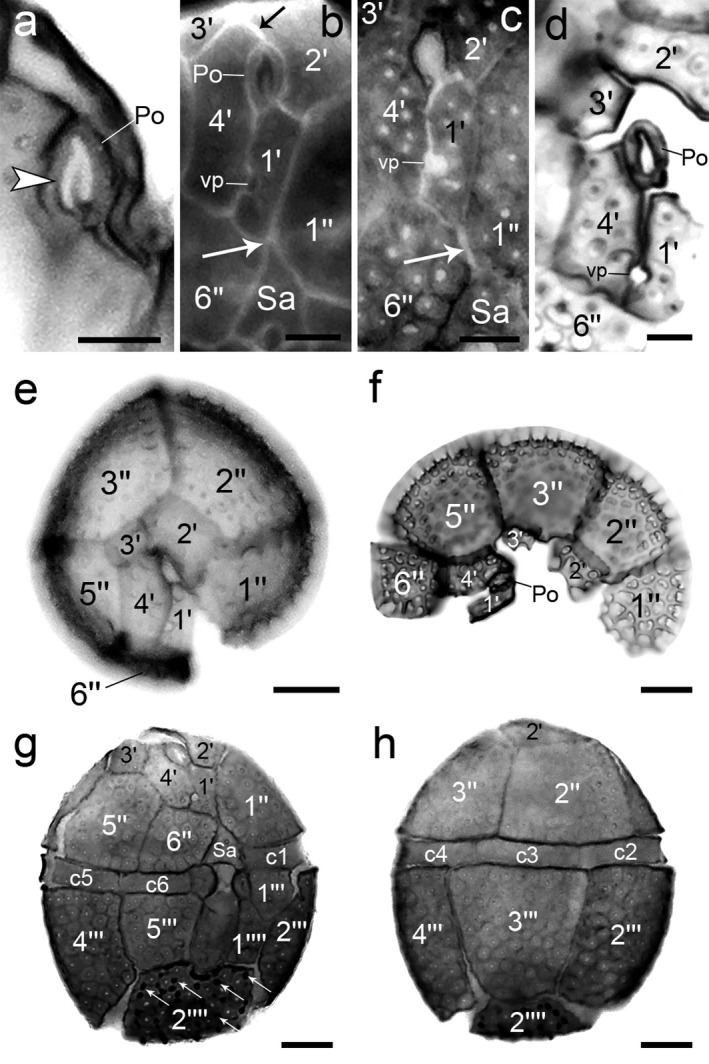
Light microscopy images of *Ceratocorys mariaovidiorum* sp. nov. cells stained with Calcofluor white. (a) Po plate with a λ‐shaped pore (arrowhead). (b) Plate 1′ contacting the Po and Sa plates through a suture and a point (white arrow), respectively, and separation of the Po and 3′ plates through a suture (black arrow). (c) Plate 6″ contacting Sa (arrow). (d) Dissection of apical plates. (e) Epithecal plates. (f) Dissection of epithecal plates. (g) Flattened cell in ventral view (arrows indicate small spines in 2′′′′). (h) Flattened cell in dorsal view. Scale bar: 5 μm (a–d), 10 μm (e–h). vp, ventral pore. The equivalence of the plate names used in this study and plate names strictly following the Kofoid system showed in Figures 1 and 2, but are not shown here in order to keep the images as clear as possible.

**Figure 4:**
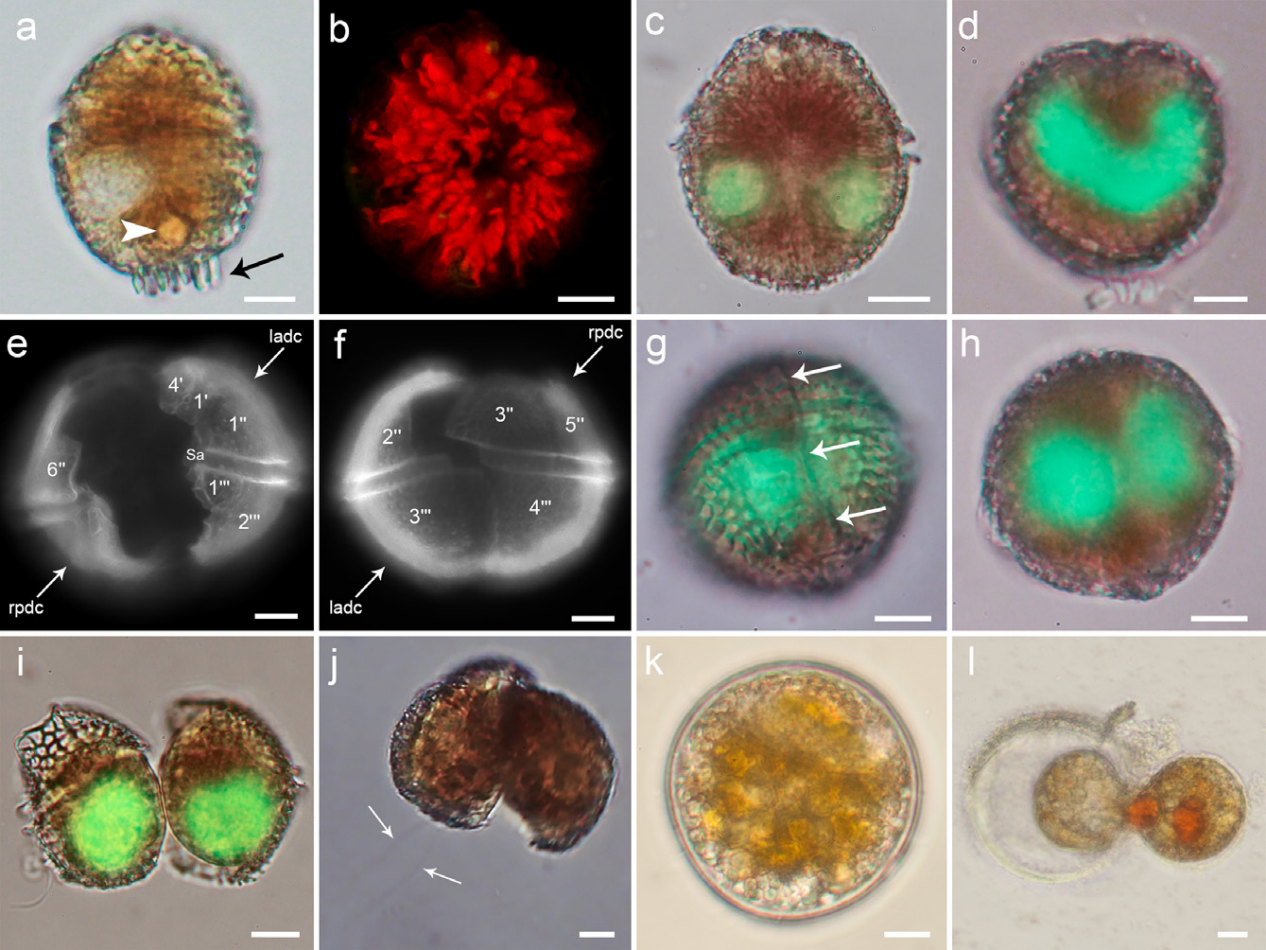
Light microscopy images of *Ceratocorys mariaovidiorum* sp. nov. cells. Cells stained with SYBR Green (c, d, g–i) and Calcofluor White (e and f). (a) Cell showing a rounded body (arrowhead) and antapical spines (arrow). (b) Chloroplasts radiating from the center of the cell. (c) Cell in ventral view showing the tips of the U‐shaped nucleus. (d) Cell in antapical view showing the U‐shaped nucleus. (e) Ventral view of a dividing cell (ladc, left anterior daughter cell; rpdc, right posterior daughter cell). (f) Dorsal view of the same dividing cell as in (e) (ladc, left anterior daughter cell; rpdc, right posterior daughter cell). (g) Cell showing a fission line (arrows) before division. (h) Different focal plane of the same cell as in (g) showing two nuclei before division. (i) Recently divided cells with round nuclei. (j) Daughter cells from a planozygote division (arrows, two longitudinal flagella). (k) Cyst‐like cell. (l) Cell hatching from a cyst‐like cell. Scale bar: 10 μm. The equivalence of the plate names used in this study and plate names strictly following the Kofoid system showed in Figures 1 and 2, but are not shown here in order to keep the images as clear as possible. [Color figure can be viewed at wileyonlinelibrary.com]

In this article, a relaxed Kofoid nomenclature system that recognizes homologies of plates of other gonyaulacoid genera was used (Fensome et al. 1993, Paez‐Reyes and Head 2013). The plate, which, following the Kofoid system, should be named an anterior intercalary plate (1a), was considered here as the homologue of the third apical plate of other gonyaulacoids because it can contact the apical pore plate (Po), and hence is named 3′ (Table 1). *Ceratocorys* has five precingular plates (Table 1), but the last plate of the series of precingular plates was named 6″ instead of 5″, as it is considered to be homologous to that plate of other Gonyaulacales. The equivalence of the plate names used here and plate names strictly following the Kofoid system are given in Table 1 and shown between brackets in Figures 1 and 2, but not in Figures 3 and 4, to keep the images as clear as possible.

**Table 1:** Plate nomenclature used in this study and that used by Sala‐Pérez et al. (2016)

This study		*Protoceratium reticulatum* (Sala‐Pérez et al. 2016)	
Po	Apical pore plate	Po	Apical pore plate
1′	First apical	1′	First apical
2′	Second apical	2′	Second apical
3′	Third apical	1a	First intercalary
4′	Fourth apical	3′	Third apical
1″	First precingular	1″	First precingular
2″	Second precingular	2″	Second precingular
3″	Third precingular	3″	Third precingular
4″	Missing	4″	Fourth precingular
5″	Fifth precingular	5″	Fifth precingular
6″	Sixth precingular	6″	Sixth precingular
1‴	First postcingular	1‴	First postcingular
2‴	Second postcingular	2‴	Second postcingular
3‴	Third postcingular	3‴	Third postcingular
4‴	Fourth postcingular	4‴	Fourth postcingular
5‴	Fifth postcingular	5‴	Fifth postcingular
1′′′′	First antapical	1′′′′	First antapical
2′′′′	Second antapical	2′′′′	Second antapical
Sa	Sulcal anterior		
Ssa	Left sulcal anterior		
Sda	Right sulcal anterior		
Saca	Accesory sulcal anterior		
Ssp	Left sulcal posterior		
Sdp	Right sulcal posterior		
Sp	Sulcal posterior		

### Phylogenetic analysis

The internal transcribed spacer (ITS) and 5.8SrDNA sequences of the studied strains and those of other related taxa included in this study were obtained from the GenBank database and aligned using MEGA 7. Uncorrected genetic *p*‐distances were calculated between *Protoceratium reticulatum*,* Ceratocorys horrida,* and *C. mariaovidiorum* sequences across the full ITS and 5.8SrDNA (except two nucleotides at the end of ITS‐2 due to the shorter EU927577 sequence of *Ceratocorys horrida*). Sequences of CCMP404 and CCMP1720 were only included if the full length of ITS/5.8SrDNA was available. Several sequences were retrieved in GenBank for both strains but, despite being identical, they were kept in the final alignment. A sequence from *Karenia brevis* was used as an outgroup. The final alignment for the ITS phylogeny consisted of 546 positions. The phylogenetic relationships were determined according to the maximum likelihood method using MEGA 7 and the bayesian inference method with a general time‐reversible model from MrBayes v.3.2 (Huelsenbeck and Ronquist 2001). The model selection tool in MEGA 7 selected Tamura and Nei (TN93) as the best model (Tamura and Nei 1993), with a gamma‐shaped parameter (*y* = 2) and proportion of invariable sites (I = 7%).

Bayesian phylogenetic inference and, in this case, the substitution models were obtained by sampling across the entire general time‐reversible model space following the procedure described in the MrBayes v.3.2 manual. The program parameters were statefreqpr = dirichlet (1,1,1,1), nst = mixed, rates = gamma. The phylogenetic analyses involved two parallel analyses, each with four chains. Starting trees for each chain were selected randomly using the default values for the MrBayes program. The corresponding number of unique site patterns was 513. The number of generations used in these analyses was 1,000,000. Posterior probabilities were calculated from every 100th tree sampled after log‐likelihood stabilization (the “burn‐in” phase). All final split frequencies were <0.02. The two methods rendered very similar topologies and the phylogenetic tree was represented using the bayesian inference results, with bootstrap values from the maximum likelihood method (*n* = 1,000 replicates) and posterior probabilities from the bayesian inference method.

### Extraction and analysis of YTXs

Cultures of strains CCMP404 and CCMP1720 were harvested for yessotoxin (YTX) determination with exponentially growing vegetative cells. Before the extraction of toxins, an aliquot of each culture was collected and fixed with Lugol solution to determine the cell density by light microscopy using a Sedgewick‐Rafter counting chamber. Culture volumes of 590 mL (5,824 cell · mL^−1^) for CCMP404 and 610 mL (6,996 cell · mL^−1^) for CCMP1720 were filtered through GF/C glass microfiber filters (47 mm diameter; Whatman, Maidstone, UK). The cells in the filter were extracted with 1.5 mL of methanol, sonicated for 1 min at 50 W with a sonication probe and centrifuged for 10 min at 4,620*g* and 5°C. The supernatant was removed and the pellet was extracted again with 1.5 mL of methanol following the same process. The two supernatants were combined and the final volume was 3 mL. The extracts were kept at −20°C until chemical analyses, at which time they were tempered and subsequently filtered through 0.45‐μm PTFE syringe filters.

YTX determination was performed by liquid chromatography coupled to high‐resolution mass spectrometry (LC–HRMS) on a Dionex Ultimate 3000 LC system (Thermo Fisher Scientific) coupled to an Exactive mass spectrometer (Thermo Fisher Scientific, Bremen, Germany) equipped with an Orbitrap mass analyzer and a heated electrospray source (H–ESI II). Nitrogen (purity >99.999%) was used as the sheath gas, auxiliary gas, and collision gas. The instrument was calibrated daily in positive and negative ion modes. Mass acquisition was performed in negative ion mode without and with all ion fragmentation (AIF) with a high‐energy collisional dissociation of 40 eV. The mass range was *m/z* 500–2,000 in full‐scan and *m/z* 500–1,500 in AIF mode.

Chromatographic conditions were according to the Standardized Operating Procedure validated by the European Union Reference Laboratory for Marine Biotoxins (EURLMB 2011). The separation column was a Gemini NX C18 (100 × 2 mm, 3 μm) maintained at 40°C. Mobile phase A consisted of water containing 0.05% ammonia and mobile phase B of acetonitrile/mobile phase A (90:10 v/v). Linear gradient elution was pumped at a flow rate of 400 μL · min^−1^ and started at 25% B. From 1.5 to 7.5 min it increased from 25% to 95% B where it was held for 6.5 min. The initial conditions of 25% B were restored in 3 min, and then maintained for 2 min to allow column equilibration. The total run time was 19 min. For YTX identification, a YTX standard purchased from the Institute of Environmental Science and Research Limited (New Zealand) was used.

## Results

**Figure 5:**
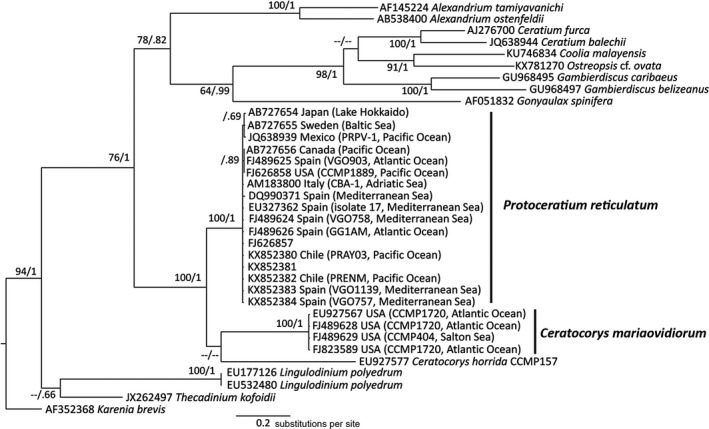
Phylogenetic relationships among *Ceratocorys mariaovidiorum* sp. nov., *Protoceratium reticulatum* strains and related taxa based on internal transcribed spacer/5.8SrDNA sequences. The phylogenetic tree was constructed using the Bayesian inference method. Numbers at the branches indicate the percentage of bootstrap support (*n* = 1,000) and the posterior probabilities based on the bayesian inference method. Bootstrap values <60% and probabilities <0.6 are denoted by hyphens.

***Ceratocorys mariaovidiorum*** P. Salgado, S. Fraga, F. Rodríguez, P. Riobó et. I. Bravo **sp. nov.** (Figs. 1, 2, 3, 4, 5);

*Holotype*: SEM‐stub (designation CEDiT2017H67) deposited at the Senckenberg Research Institute and Natural History Museum, Center of Excellence for Dinophyte Taxonomy, Germany, from which Figure 1, a–i from strain CCMP404 was obtained. The strain CCMP404 is barcoded in GenBank with nucleotide sequences of the nuclear ribosomal ITS1‐5.8S‐ITS2 (GenBank FJ489629 and EU532485), and LSU (GenBank FJ489623 and EU532476). The clonal culture strain is deposited at the National Center for Marine Algae and Microbiota, ME USA;

*Type locality*: Salton Sea, CA, USA (strain CCMP404);

*Habitat*: Hypersaline lake and marine;

*Etymology*: The word *mariaovidiorum* is composed in its first part by maria of the name María and in its second part by ovidiorum, of the name Ovidio, and indicates the parents’ names of the first author of this work;

*Description*: Cells have a length of 33.8–50.1 μm and a width of 31.3–47.3 μm. They are globular in ventral and dorsal views, and almost round in apical and antapical views, without horns or large spines, although the second antapical plate (2′′′′) may have small spines. The epitheca is almost as long as the hypotheca. The cingulum is excavated with its right end displaced posteriorly one cingular width. The plate formula is Po, 4′, 0a, 5″, 6c, ~7s, 5‴, 0p, 2′′′′. The Po is oval with a λ‐shaped pore. The first apical plate (1′) is narrow, with a ventral pore on the right anterior side. This plate contacts the Po, while the contact with the anterior sulcal plate (Sa) is slight or absent. The third apical plate (3′) is four‐sided. The second (2″) and third (3″) precingular plates are of similar size. The third, fifth (5″), and sixth (6″) precingular plates are five‐sided. The fourth precingular plate (4″) of other Gonyaulacales is absent. The hypotheca is composed of five postcingular and two antapical plates. Thecal plates are thick and strongly reticulated, with one pore within each reticule;

*Distribution*:* Ceratocorys mariaovidiorum* was isolated from Salton Sea (strain CCMP404), CA, USA and from Biscayne Bay (strain CCMP1720), Miami, FL, USA. It has also been reported (identified as *P. reticulatum*) from the eastern of Gulf of Mexico (Steidinger and Williams 1970), the Indian River Lagoon, FL, USA (Hargraves 2017), and the Belize coast (Faust et al. 2005);

*Morphology*: The cells are almost spherical in apical and antapical views (Fig. 1, c and h). They have an epitheca almost as long as the hypotheca (Figs. 1a and 2a). Cells of strain CCMP404 range from 36.5 to 47.1 μm in length (mean and SD of 42.4 ± 2.6 μm length, *n* = 30) and from 34 to 45.3 μm in width (39.5 ± 2.6 μm width, *n* = 30). Strain CCMP1720 ranges from 33.8 to 50.1 μm in length (40.4 ± 3.6 μm length, *n* = 30) and from 31.3 to 47.3 μm in width (37.3 ± 3.5 μm width, *n* = 30). Cell depth in both strains is almost equal to cell width (Fig. 1, c and h). The plate formula is Po, 4′, 0a, 5″, 6c, ~7s, 5‴, 0p, 2′′′′ (Fig. 2, a–d). Thecal plates are strongly reticulated with one pore within each reticule. In the epitheca, the Po plate is oval with a λ‐shaped pore (Fig. 3a). Plate 1′ is narrow, pentagonal in shape, has a ventral pore on the right anterior side and contacts the Po through a clear suture on the anterior side, while the contact with the Sa plate is slight (Fig. 3b) or absent (Fig. 3c). Plate 1′ contacts on its left side 1″ and 2′ and on its right side 4′ and 6″ (Figs. 2c and 3b). The second apical plate (2′) is the largest of the series and contacts 1′, 1″, 2″, 3″, 3′, and 4′ (Figs. 1b; 2c; and 3e). Plate 3′ is four‐sided and contacts 2′, 3″, 5″, and 4′ (Figs. 2c and 3e). It is separated from the Po by a short suture of the contact of plates 2′ and 4′ (Figs. 2c and 3b), but it can sometimes touch the Po with an evident separation between 2′ and 4′ (Fig. 3d). Fourth apical plate (4′) is irregular and elongated (Figs. 1d; 2c; and 3e). In addition to the Po, it contacts 1′, 2′, 3′, 5″, and 6″. The series of precingular plates is composed of large plates except 6″, which is smaller than the others (Figs. 1c and 3, e and f). The first precingular plate (1″) contacts the anterior left part of Sa, 2″, 2′, and 1′ (Figs. 1, a and b and 2c). Plate 2″ is four‐sided and contacts 3″, 2′, and 1″ (Figs. 1b; 2c; and 3, e and f). Plate 3″ is five‐sided, is located in right dorsal position, grows overlapping all its neighboring plates, 5″, 3′, 2′, and 2″ (Figs. 1c and 3e), and is the keystone plate in the sense given by Fensome et al. (1993). The four‐sided plate 4″ of other gonyaulacoids is absent (Figs. 1c; 2c; and 3, e and f). Plate 5″ is five‐sided and contacts 3′, 4′, 6″, and 3″ (Figs. 1d and 2c). Plate 6″ is the smallest of the series (Figs. 2c and 3f), square in shape, and contacts Sa, 1′, 4′, and 5″ (Figs. 1a and 2c), and in some cases 1″ (Fig. 3c). The cingulum descends one width without overhanging. The cingulum and sulcus are narrow and excavated (Figs. 1a and 2a). The cingulum is composed of six plates, c1–c6 (Fig. 3, g and h), which are non‐reticulated with two rows of pores along their anterior and posterior margins (Fig. 1a). The sulcus is composed of at least seven plates, Sa, left sulcal anterior (Ssa), right sulcal anterior (Sda), accessory sulcal anterior (Saca), left sulcal posterior (Ssp), right sulcal posterior (Sdp), and sulcal posterior (Sp; Fig. 2b). In the hypotheca, the first postcingular plate (1‴) is the smallest of the series and contacts 2‴, 1′′′′, and Ssa (Figs. 1f; 2d; and 3g). In some cases, plate Ssa may be visible behind this plate (see Figs. 1a and 2b). The second (2‴), third (3‴), and fourth (4‴) postcingular plates are the largest and form the main body of the hypotheca (Figs. 1g; 2d; and 3h). The five‐sided 2‴ contacts 3‴, 2′′′′, 1′′′′, and 1‴ (Figs. 1, f–i and 2d). Plate 3‴ overlaps its neighbors 4‴, 2′′′′, and 2‴ (Figs. 1, g–i and 2d). Like 3‴, plate 4‴ is four‐sided and contacts 3‴, 2′′′′, and 5‴ (Figs. 1, e and g and 2d). The fifth postcingular plate (5‴) is almost square in shape (Figs. 1, e and f and 2d). The first antapical plate (1′′′′) forms a thin list along its right side and hides the sulcal area (Fig. 1h). Plate 2′′′′ is almost square with a notch toward the sulcus, contacts 1′′′′, 2‴, 3‴, 4‴, and 5‴ (Figs. 1, e–i and 2d), and may have small spines (Figs. 3, g and h and 4a);

The cells show numerous chloroplasts which radiate from the central part of the cell (Fig. 4b), a centrally located U‐shaped nucleus with the tips ventrally directed (Fig. 4, c and d) and, in some cases, one or two large orange bodies in the posterior part of the hypotheca (Fig. 4a).

*Life cycle*: Vegetative cells with the above‐described morphology divide asexually by desmoschisis, in which each daughter cell inherits part of the parental thecal plates (Fig. 4, e and f). The fission line is oblique, to the right of the longitudinal axis in the epitheca and to the left in the hypotheca. In this process, the right posterior daughter cell that inherits the flagellar apparatus of the mother retains both flagella (transversal and longitudinal), while the left anterior daughter cell forms them later. Before splitting, the dividing cells show the fission line and two centrally located oval nuclei (Fig. 4, g and h). Once division finishes, each recently divided cell has a round nucleus (Fig. 4i). In addition to motile cells with a globular shape, a type of cell with a strong dorso‐ventral compression was regularly present in the cultures, though in very low number. This cell type has recently been described by Salgado et al. (2018) as a benthic stage of the life cycle. Sexual stages such as gamete pairs and planozygotes were also observed in both strains cultured in L1 medium. Longitudinally, biflagellate cells (planozygotes) also divide by desmoschisis, in which the right posterior daughter cell inherits the two trailing flagella of the mother (Fig. 4j). Moreover, spherical cells with the appearance of cysts (Fig. 4k) were formed inside large, non‐motile thecate cells lying on the bottom of the culture flasks. Although their sexual or asexual nature is unknown, these cyst‐like cells had a double cell wall without processes, many transparent granules, and one or two large orange bodies. In fresh replete medium all these cells germinated (Fig. 4l) within the first 5 d after isolation, giving a normal thecate cell that divided within the first 3 d after hatching;

*Phylogenetic analyses*: The bayesian inference analysis of the ITS/5.8SrDNA region showed a clear divergence between the two strains of *Ceratocorys mariaovidiorum* used in this study and the sequences of *Protoceratium reticulatum* from isolates throughout the world (Fig. 5). Our phylogeny grouped *Ceratocorys* sequences as a separate monophyletic group from the *Protoceratium reticulatum* clade, whereas *C. horrida* and *C. mariaovidiorum* diverged as separate branches, in which the *C. mariaovidiorum* clade is slightly closer to the *P. reticulatum* clade. Uncorrected *p*‐distances across the full ITS and 5.8SrDNA fragments were slightly larger between *C. horrida* and *C. mariaovidiorum* (0.408) than those between them and *P. reticulatum* (0.379–0.387 and 0.304–0.313 for *C. horrida* and *C. mariaovidiorum* respectively). These genetic distances mainly arose from the nucleotide differences between *Ceratocorys* and *Protoceratium* in the ITS‐1 and ITS‐2 fragments (0.49–0.57 and 0.38–0.50 respectively). In comparison, *p*‐distances between *P. reticulatum* sequences ranged from 0.012 to 0.030 in the ITS‐1/ITS‐2 fragments. In turn, the 5.8SrRNA gene was much conserved between *P. reticulatum* and both *Ceratocorys* species (0.026–0.066);

*Toxin analyses*: YTX, 45 OH‐YTX, Homo YTX and 45 OH‐Homo YTX were not detected by LC–HRMS in either CCMP404 or CCMP1720. The limit of detection (LOD) for YTX with the LC–HRMS system and conditions detailed in this work was found to be 17 pg on column, which considering the number of cells used in the analysis, this implies a YTX LOD value of 0.7 fg per cell.

## Discussion

The results presented herein show that strains CCMP404 and CCMP1720, previously listed as *Protoceratium reticulatum* (Paz et al. 2007, Howard et al. 2009, Akselman et al. 2015, NCMA 2017), actually correspond to a new species of the genus *Ceratocorys*,* C. mariaovidiorum*. This new species also includes the strain CCMP1721 (NCMA 2017), isolated from Florida waters just like CCMP1720 (Scorzetti et al. 2009, NCMA 2017), which forms a clade together with strains CCMP1720 and CCMP404 in the phylogenetic tree of Akselman et al. (2015). The great morphological resemblance between *C. mariaovidiorum* and *P. reticulatum* would account for the previous misidentification, because in ventral view, even in SEM, they are hardly distinguishable, if at all. Our results classify those strains as *Ceratocorys* based on tabulation of thecal plates and phylogeny, but there are some reasons to be cautions about this issue (see below in the *Phylogeny* section). The results also reveal that *C. mariaovidiorum* is the species most different from other ceratocoryoids as well as the species most alik to the genus *Protoceratium*. In addition to having smaller cells, *C. mariaovidiorum* differs mainly in shape from other members of the genus. In general, they have an angular body, with the exception of *C. gourretii*, which is subspherical to ovoid, *C. horrida* being the most angular (Graham 1942), while *C. mariaovidiorum* is almost spherical. According to Fensome et al. (1993), *Ceratocorys* has a distinctive general pattern: the episome is flat and forms only about one‐fifth of the total length of the central body, and the hyposome consists of the deep lower fourth‐fifths of the central body. Graham (1942) mentioned, however, that the girdle is placed almost equatorially in *C. armata* and *C. reticulata*, which agrees more with *C. mariaovidiorum*.

### Morphology

The plate tabulation of *Ceratocorys mariaovidiorum* was based herein on plate homologies of other gonyaulacoid genera (Fensome et al. 1993, Paez‐Reyes and Head 2013), giving a thecal plate formula Po, 4′, 0a, 5″, 6c, ~7s, 5‴, 0p, 2′′′′. However, a different interpretation of the 3′ plate can be made when it is not in contact with Po (1a according to the Kofoid system), resulting in a plate formula Po, 3′, 1a, 5″, 6c, ~7s, 5‴, 0p, 2′′′′. The contact variability between the 1a and Po plates has also been observed in *P. reticulatum* cells coming from cultures and wild populations (see Woloszynska 1928, Hansen et al. 1996/97, Sala‐Pérez et al. 2016, Salgado et al. 2017), so it is quite likely that plates 1a and 3′ are homologous in both *P. reticulatum* and *C. mariaovidiorum*. According to Graham (1942), the epitheca of *Ceratocorys* has 4′ and 0a, which is in line with our interpretation of epithecal plate tabulation, but this finding was refuted by Balech (1988) who indicated that the third apical is an intercalary plate (1a) because he observed it always separated from the Po. The contact between these two plates in *C. mariaovidiorum* agrees with the observation by Taylor (1976) for the genus *Ceratocorys*, which is why he thought this plate could also be considered as apical.

The thecal surface of *Ceratocorys mariaovidiorum* is very similar to that described for *P. reticulatum* (Balech 1988). The strong reticulation in both species often prevents a clear assessment of the number and orientation of plates (Hargraves and Maranda 2002), which further complicates the identification of each species when a staining such as Calcofluor is not used or the plates are not dissected. The main morphological differences between *C. mariaovidiorum* and *P. reticulatum* are the number of precingular plates—five in the former and six in the later—and the amount of apparent contact of Sa with 1′, which is considerable in *P. reticulatum*, whereas in *C. mariaovidiorum* it is slight or absent. Fensome et al. (1993) mentioned that the contact between plates 6″ and 1″, with subsequent lack of contact between Sa and 1′, is considered a key character of the genus *Ceratocorys*. Additionally, these two species differ in the shape of the apical pore, which is λ‐shaped in *C. mariaovidiorum* and is a narrow unbranched slit in *P. reticulatum* (see Fig. 3, a and b in the present paper, fig. 7 in Hansen et al. 1996/97, and fig. 2, n and o in Sala‐Pérez et al. 2016). Interestingly, the Po plate of *P. reticulatum* shown by Hansen et al. (1996/97) and Sala‐Pérez et al. (2016) appears to be similar to that of *C. armata* (see plate 3G in Steidinger and Tangen 1997), but apparently different from that of *Gonyaulax grindleyi* (= *P. reticulatum*) shown in Plate 4D by Steidinger and Tangen (1997), which according to Steidinger has two portions (K.A. Steidinger, pers. comm.). What makes the scenario even more interesting is that the Po of *C. mariaovidiorum* would be different from that of *C. armata*. The lack of morphological information on the Po plate of *C. horrida* (the type species of *Ceratocorys*) makes it difficult to obtain conclusions on this subject. All these findings need to be elucidated by studies with more isolates and wild populations from the type localities of *P. reticulatum* (including *G. grindleyi*), *C. mariaovidiorum* and *C. horrida*, in order to study the plates, the Po, the morphometrics, and the gene sequences. Nevertheless, variability in the shape of the Po has been shown for *Pyrodinium bahamense* (Mertens et al. 2015), indicating that situations like these may not be so rare and that the variability in the Po must be studied thoroughly.

Another morphological feature that makes a difference between *Ceratocorys mariaovidiorum* and *Protoceratium reticulatum* is the presence of small antapical spines in *C. mariaovidiorum*, although this ornamentation is a variable trait that is not expressed in the same way in all individuals. Some cells had small spines, others had larger ones that can be clearly seen in light microscopy, while yet others did not seem to have them (compare Figs. 3g and 4, a and c). The number and size of spines on plate 2′′′′ is a main characteristic feature of the genus *Ceratocorys* (Carbonell‐Moore 1996, Gómez et al. 2011) and such spines have not been reported in *P. reticulatum*. Currently, the only two species that do not develop spines are *C. anacantha* and *C. grahamii* (Carbonell‐Moore 1996). Other members, *C. skogsbergii*,* C. armata*,* C. reticulata*, and *C. aultii* have short hypothecal spines (see figs. 53–56, and 58 in Graham 1942). Environmental conditions in nature and experimental conditions in cultures (e.g., turbulence, salinity, temperature, light irradiation, and photoperiod cycle) may influence the development of the processes in dinoflagellate cysts or vegetative cells. The length and number of processes in the *Lingulodinium polyedra* cyst is related to the summer salinity and temperature at a water depth of 30 m (Mertens et al. 2009). *Tripos ranipes*, a species characterized by having fingers at the end of the horns, loses them during the night and recovers them the following day (Pizay et al. 2009). In laboratory culture of *C. horrida*, it was observed that the shape and length of the spines depends on turbulence. Fluid motion inhibits the growth of large spines, which may be reversible when normal conditions are reestablished (Zirbel et al. 2000). Those results suggest that the morphology and even the presence of the spines in *C. mariaovidiorum* can be a trait modulated by external characteristics in addition to the genetic ones.

### Phylogeny

Phylogenetic analyses support the classification of *Ceratocorys mariaovidiorum* as a new species of *Ceratocorys*. However, a single monophyletic *Ceratocorys* clade was not robustly defined. Litaker et al. (2007) indicated that uncorrected *p*‐distances for ITS/5.8SrDNA between 81 dinoflagellate species from the same genera (*n* = 14) ranged from 0.042 to 0.580. This result led them to propose that *P* ≥ 0.04 could be used to delineate most free‐living dinoflagellate species. Based on their results, *Protoceratium reticulatum* and *C. mariaovidiorum* clades must represent different species. The *p*‐distances of ITS/5.8SrDNA between *P. reticulatum*,* C. mariaovidiorum* and *C. horrida* (0.304–0.387) fall into the upper range of variability within a genus (Litaker et al. 2007), but the species boundaries reported by these authors were very different (as low as 0.234–0.255 for *Prorocentrum* and *Scrippsiella*) reflecting the evolutionary history of each genus. Therefore, based on current taxonomic criteria and the lack of additional sequences of *Ceratocorys*, integrating *C. mariaovidiorum* into the genus *Ceratocorys* was considered the most appropriate action at this time. The emergence of a new species was already suggested previously by Akselman et al. (2015), who mentioned that the genetic divergence based on D1/D2 regions of LSUrRNA between two *P. reticulatum* clades (Clade A and Clade B) must correspond to separate evolving units. Clade B comprised the *C. mariaovidiorum* strains in the present study (CCMP404 and CCMP1720), plus CCMP1721. Those authors also reported that the genetic distance between the clades was enough to consider the two groups as different species. Our ITS‐based phylogeny confirmed their results. Further analyses (e.g., concatenated ribosomal gene phylogenies) including new sequences of other *Ceratocorys* species could be very helpful to clarify the phylogenetic relationships within this genus and toward *Protoceratium*. The close relationship between the genus *Protoceratium* and *Ceratocorys* has already been pointed out by Saldarriaga et al. (2004), who discussed the differences between molecular analyses and taxonomic position based on morphology (i.e., Fensome et al. 1993) of *Protoceratium*, declaring that SSU‐based phylogenies place *Protoceratium* closer to *Ceratocorys* than to *Lingulodinium*,* Gonyaulax*, and *Amylax*. Our data also corroborate the taxonomical position of the *Ceratocorys* genus in the order Gonyaulacales because molecular phylogeny has shown that *Ceratocorys* and *Protoceratium* have a common ancestor (Gómez et al. 2011). In a revised classification of dinoflagellate taxonomy, based on the morphological traits of *Ceratocorys* and the scarce molecular phylogenetic data, Hoppenrath (2016) placed this genus as “incertae sedis” within Asymmetricomorpha but outside gonyaulacoids. However, the finding of *C. mariaovidiorum* close to *Protoceratium* but belonging to *Ceratocorys* rather indicates that this genus should be considered inside the order Gonyaulacales.

### Distribution

Thus far, the distribution of *Ceratocorys mariaovidiorum* has to be deduced from the locations where strains now recognized as this species were isolated. Strain CCMP404 comes from the Salton Sea, a hypersaline eutrophic lake located in southeastern CA, USA, and strains CCMP1720 and CCMP1721 come from Biscayne Bay in FL, USA (Scorzetti et al. 2009, NCMA 2017). However, there are other cases in the literature that deserve to be discussed herein because in our opinion they should be considered as *C. mariaovidiorum* instead of *Protoceratium reticulatum*. Hargraves (2017; see figs. 5 and 6) showed SEM microphotographs of cells and an epitheca of *C. mariaovidiorum* (identified as *P. reticulatum*) from the Indian River Lagoon in FL, USA. *Ceratocorys mariaovidiorum* (as *Gonyaulax grindleyi*) from two Belizean coral‐reef mangrove lagoons in the Caribbean Sea can be deduced from Faust et al. (2005) (see figs. 2–5, p. 121), who show an epitheca with five precingular plates and an oval Po plate. Also from Belize (The Lair, Twin Cays and South Water Cay), cells with five precingular plates corresponding to *C. mariaovidiorum* (as *Gonyaulax grindleyi*) have been reported (EOL 2017). According to Steidinger and Williams (1970), *P. reticulatum* was observed (fig 140c) off the east coast of the Gulf of Mexico during the Hourglass expedition. In that report the authors show an optically reversed dissected epitheca, which, according to the interpretation adopted herein, has the same five precingular and three apical plates as the one shown in *C. mariaovidiorum* (Fig. 3f) with the exception that in the former image plates 1′ and Po do not appear. Although both species are found in the area, Steidinger believes that much of what has been recorded as *P. reticulatum* in the eastern Gulf of Mexico is *C. mariaovidiorum* because of the shape of the Po plate (K.A. Steidinger, pers. comm.). On the other hand, a strong suspicion of the presence of *C. mariaovidiorum*, in addition to that of *P. reticulatum*, in the warm waters of Kuwait has also been indicated (M. Saburova, pers. comm.).

So far, *Ceratocorys mariaovidiorum* has been identified only in warm waters (the Gulf of Mexico, the Caribbean Sea, the east coast of Florida, and the Salton Sea), while *Protoceratium reticulatum* has a wider latitudinal distribution, being considered mainly a boreal species (Okolodkov 2005), although it has also been reported from warm waters (strain FIU20/CCMP2776 in Scorzetti et al. 2009).

### Life cycle

The pattern of asexual division of *Ceratocorys mariaovidiorum* (desmoschisis) matches that of *Protoceratium reticulatum* (Hansen et al. 1996/97, Salgado et al. 2017) and other species of *Ceratocorys* (Graham 1942, Fensome et al. 1993). As described for vegetative cells, external morphological similarities of both asexual stages (dividing and recently divided cells) and sexual stages (gamete pairs, planozygotes and dividing planozygotes) between these species were observed. In addition, formation of benthic flattened cells from vegetative ones is also shared by both species. The formation process and morphological features are described in detail by Salgado et al. (2018). On the other hand, no cysts resembling the typical spiny resting cysts of *P. reticulatum* appeared in the cultures of *C. mariaovidiorum*. The cyst‐like cells formed in the cultures showed a thick wall but did not show processes. They were formed in very low numbers and, although they germinated in few days, more studies are needed to determine their significance and role in the life strategy of the species. Taking into account that for *P. reticulatum* only four out of 66 crosses formed resting cysts (Salgado et al. 2017), the only two strains of *C. mariaovidiorum* available were too few for studying resting cyst production.

### Toxins

The LC–HRMS analyses showed that *Ceratocorys mariaovidiorum* is not a producer of YTXs. Our results agree with those of Paz et al. (2007), who reported the lack of this toxin type in strains CCMP404, CCMP1720, and CCMP1721 from the USA when analyzed by LC–FLD. In addition, Reifel et al. (2002) showed that *Protoceratium reticulatum* from the Salton Sea, where the *C. mariaovidiorum* strain CCMP404 was isolated, showed no toxicity in mouse and brine shrimp assays and a proton nuclear magnetic resonance spectra did not show the presence of structures similar to those of algal toxins, suggesting that the species probably corresponds to *C. mariaovidiorum*. The confirmation of non‐production of YTX in *C. mariaovidiorum* reveals another important difference from *P. reticulatum*, which is known as a YTX producer worldwide (e.g., Ciminiello et al. 2003, Paz et al. 2004, Eiki et al. 2005, Álvarez et al. 2011, Akselman et al. 2015, Sala‐Pérez et al. 2016). As far as we know, only two reports (Satake et al. 1999, Rhodes et al. 2006) have indicated the lack of toxins in *P. reticulatum* strains. First, Rhodes et al. (2006) analyzed two New Zealand strains, of which one (CAWD40) produced YTXs and the other (CAWD127) did not. Both strains fell in the same clade (Clade A) of the phylogenetic tree of Akselman et al. (2015). Second, Satake et al. (1999) reported one YTX‐producing isolate from Yamada Bay and another non‐toxic strain from Harima Nada, but no genetic information is available on these Japanese strains. Unlike Yamada Bay, Harima Nada is in an area of warm waters, so the isolate coming from this locality might correspond to *Ceratocorys mariaovidiorum*.

In conclusion, it is thus not clear whether the presence of YTX is a stable and species‐specific trait of *Protoceratium reticulatum*. Likewise, more studies on the lack of YTX in *Ceratocorys mariaovidiorum* are needed using more strains from other geographical origins.
